# Supplementary material for: Strongly enhanced dynamics of a charged Rouse dimer by an external magnetic field
Source: PNAS Nexus. 2022 Jul 23;1(3):pgac119. doi: 10.1093/pnasnexus/pgac119 (PMC9896929; doi:10.1093/pnasnexus/pgac119)
Supplement: pgac119_Supplemental_File [file pgac119_supplemental_file.pdf]

Supplementary Information for

Strongly enhanced dynamics of a charged Rouse dimer by an external magnetic field

Rushikesh Shinde, Jens Uwe Sommer, Hartmut Löwen, Abhinav Sharma

Abhinav Sharma.
E-mail: sharma@ipfdd.de

This PDF file includes:

Supplementary text
Figs. S1 to S3
SI References

Supporting Information Text

1. Diffusion under Lorentz force

Consider a single particle carrying a charge q in a uniform magnetic field $\vec{B}$. Let $\vec{n}$ be the unit vector along the magnetic field and B be the magnitude. Due to the magnetic field, the particle is subjected to the Lorentz force $q\vec{v} \times \vec{B}$ where $\vec{v}$ is its velocity. The dynamics of the particle are described by the following Langevin equation:

$$m\frac{d\vec{v}(t)}{dt} = -\mathbf{\Gamma}\vec{v}(t) + \vec{\chi}(t). \quad [1]$$

$\mathbf{\Gamma} = \gamma\mathbf{I} + qB\mathbf{N}$ is an inverse mobility tensor where $\mathbf{I}$ is identity matrix and $\mathbf{N}$ is an anti-symmetric tensor with elements $N_{xy} = -\epsilon_{xyz}n_z$, where ϵ is the Levi-Civita symbol in three dimensions and n_z is the z-th component of $\vec{n}$. $\vec{\chi}(t)$ is a white Gaussian noise with zero mean and time correlation $\langle\vec{\chi}(t)\vec{\chi}(s)\rangle = 2\gamma k_B T\mathbf{I}\delta(t-s)$, where k_B is the Boltzmann constant and T is the temperature.

We are interested in the overdamped dynamics. Simply ignoring the inertial term ($m \to 0$) in the Langevin equation (Eq. (1)) does not yield the correct overdamped equation of motion (1). Chun et al showed (2), after taking the appropriate low mass limit the governing Langevin equation reads

$$\mathbf{\Gamma}\frac{d\vec{r}}{dt} = \vec{\eta}(t), \quad [2]$$

where the noise $\vec{\eta}(t)$ in Eq. (2) is a non-white Gaussian noise with zero mean. The time correlation function for the noise is given as follows:

$$\left\langle\vec{\eta}(t)\vec{\eta}^T(s)\right\rangle = k_B T\left(\mathbf{\Gamma}^T\,\delta_+(t-s) + \mathbf{\Gamma}\,\delta_-(t-s)\right), \quad [3]$$

where $\delta_\pm(u)$ are modified Dirac δ functions such that $\delta_+(t) = \delta(t-\epsilon$ where ϵ is an infinitesimally small positive number. Likewise, $\delta_-(t) = \delta(t+\epsilon)$. These modified functions are related to each other as $\delta_+(t) = \delta_-(-t)$. Also note that $\int_0^\infty du\delta_+(u) = \int_{-\infty}^0 du\delta_-(u) = 1$ and $\int_{-\infty}^0 du\delta_+(u) = \int_0^\infty \delta_-(u) = 0$.

From a computational perspective, Eq. (3) is not particularly useful as it is currently not known how to simulate the noise due to its non-white nature. Therefore, one uses Eq. (1) with a small mass to study the overdamped dynamics. Despite this, one can obtain the Fokker Planck equation (Diffusion equation) for the position degrees of freedom (1). Because the noise is non-white, it has been previously argued that equation governing the phase space dynamics can not be called a Fokker-Planck Equation (2). Nevertheless, since the Brownian noise is still Gaussian so we only have to consider the first two moments of the probability distribution in order to exactly capture the evolution of the probability density function. Hence we think it is still appropriate to call the governing continuity equation the Fokker Planck equation (FPE) (3). This FPE picks up the following diffusion tensor:

$$\begin{aligned}\mathbf{D} &= k_B T\mathbf{\Gamma}^{-1}, \\ &= \frac{k_B T}{\gamma}\left[\left(\mathbf{I} + \frac{\kappa^2}{1+\kappa^2}\mathbf{N}^2\right) - \frac{\kappa}{1+\kappa^2}\mathbf{N}\right], \\ &= \mathbf{D}_s + \mathbf{D}_a,\end{aligned} \quad [4]$$

where $\kappa = qB/\gamma$ is a dimensionless constant. This has been recently referred to as the diffusive Hall-effect parameter. It is the ratio of the Lorentz force acting on the particle to the frictional drag force. $\mathbf{D}_s$ and $\mathbf{D}_a$ are the symmetric and antisymmetric parts of the diffusion tensor. The antisymmetric tensor $\mathbf{D}_a$ gives rise to fluxes that are perpendicular to the density gradients. Being rotational in nature, these fluxes have zero divergence and hence do not contribute to the dynamics of the position distribution of the particle. They can therefore be ignored when one is interested only in the position statistics. However, a disregard of the Lorentz fluxes leads to erroneous heat dissipation and entropy production in the system (1, 2, 4).

2. MSD calculation

Consider a Rouse dimer consisting of two charged particles q_1 and q_2 with position vectors denoted by $\vec{r}_1$ and $\vec{r}_2$ connected to each other via a harmonic spring of stiffness k. In addition to the Lorentz force due to a magnetic field $\vec{B} = B\vec{n}$, the two particles are subjected to non-white Gaussian noises (Eq. (3)) denoted by $\vec{\eta}_1$ and $\vec{\eta}_2$. The Langevin equations for the two particles are given as

$$\mathbf{\Gamma}_1\frac{d\vec{r}_1}{dt} = -k(\vec{r}_1 - \vec{r}_2) + \vec{\eta}_1(t), \quad [5]$$

$$\mathbf{\Gamma}_2\frac{d\vec{r}_2}{dt} = -k(\vec{r}_2 - \vec{r}_1) + \vec{\eta}_2(t), \quad [6]$$

where $\mathbf{\Gamma}_1 = \gamma\mathbf{I} + q_1 B\mathbf{N}$ and $\mathbf{\Gamma}_2 = \gamma\mathbf{I} + q_2 B\mathbf{N}$.

To get Langevin equations with statistically independent noises, we transform the coordinates as follows:

$$\vec{r}_+ = \frac{\mathbf{\Gamma}_1\vec{r}_1 + \mathbf{\Gamma}_2\vec{r}_2}{2\gamma}, \qquad \vec{r}_- = \frac{\mathbf{\Gamma}_1\vec{r}_1 - \mathbf{\Gamma}_2\vec{r}_2}{\gamma}. \quad [7]$$

For the case of a dimer holding equal charges, i.e., $q_1 = q_2 = q$, the transformed coordinates $\vec{r}_+$ and $\vec{r}_-$ reduce to $\vec{R} = (\vec{r}_1 + \vec{r}_2)/2$ and $\vec{r} = \vec{r}_1 - \vec{r}_2$, respectively. In this case, the joint probability distribution of the center of mass ($\vec{R}$) and bond vector ($\vec{r}$) is

$$P(\vec{R}, \vec{r}, t) \propto \exp\Big[- \frac{1}{2D_0\, \tau_s\, (1 - e^{-\frac{4t}{\tau_m}})} |\vec{r}|^2 - \frac{1+\kappa^2}{2D_0 t} |\vec{R}|^2 \Big]. \quad [8]$$

If the dimer carries different charges, the dynamics of the bond vector and the center of mass get coupled to each other. In the special case of $q_1 = -q_2 = q$, Langevin equations for the transformed coordinates $\vec{r}_+$ and $\vec{r}_-$ read

$$\frac{d\vec{r}_+}{dt} = \frac{1}{2\gamma} \vec{\xi}(t), \quad \frac{d\vec{r}_-}{dt} = \frac{1}{\gamma} \Big[\vec{\xi}(t) - \frac{2k}{k_B T} \mathbf{D}_s (2\mathbf{D}_s^{-1} \mathbf{D}_a \vec{r}_+ - \vec{r}_-) \Big]. \quad [9]$$

where $\vec{\xi}(t)$ is a white Gaussian noise with the properties

$$\big\langle \vec{\xi}(t) \big\rangle = 0, \qquad \big\langle \vec{\xi}(t) \vec{\xi}^T(s) \big\rangle = 4k_B T \gamma \mathbf{I} \delta(t - s). \quad [10]$$

Due to the white Gaussian nature of the noise, Eq. (9) can be simulated using conventional stochastic simulation methods. We note that these equations constitute a multivariate Ornstein Uhlenbeck process. The joint probability distribution function is, therefore, Gaussian and reads

$$P = P(\vec{r}_- | \vec{r}_+, t) P(\vec{r}_+, t). \quad [11]$$

Here,

$$P(\vec{r}_+, t) \propto \exp\Big[- \frac{1}{4Dt} |\vec{r}_+|^2 \Big], \quad [12a]$$

$$P(\vec{r}_- | \vec{r}_+, t) \propto \exp\Big[- \frac{1}{2} (\vec{r}_- - \vec{\mu}_t)^T \mathbf{\Sigma}_t^{-1} (\vec{r}_- - \vec{\mu}_t) \Big], \quad [12b]$$

where $D = k_B T / 2\gamma$ is the diffusion constant of the center of mass of an uncharged dimer. $\vec{\mu}_t$ is $2k[\mathbf{I} - \exp(-2\mathbf{I}t/\tau_m)]\mathbf{N}\vec{r}_+$ and the matrix $\mathbf{\Sigma}_t = 2D\tau_m[\mathbf{I} - \exp(-2\mathbf{I}t/\tau_m)]$. The joint probability distribution of the center of mass and bond vector can be obtained from Eq. (12) via a straightforward albeit tedious calculation.

The above considerations serve to highlight the Lorentz force induced coupling between different degrees of freedom. Below we describe an alternative way to obtain the mean squared displacement (MSD) of the center of mass from the Fokker-Planck equation. The FPE for the time evolution of probability density $P \equiv P(\vec{r}_1, \vec{r}_2, t)$ is given as

$$\frac{\partial P}{\partial t} = \nabla_1 \cdot \Big[\vec{\vartheta}_1 P \Big] + \nabla_2 \cdot \Big[\vec{\vartheta}_2 P \Big], \quad [13]$$

where $\vec{\vartheta}_i$ is the velocity of the particle indexed i and $\vec{\vartheta}_i P$ is the corresponding probability flux. To calculate $\vec{\vartheta}_i$, we write down the force balance equation for each particle. In overdamped regime, sum of all forces and torques acting on each particle must be zero. This gives

$$\vec{F}_{Br} + \vec{F}_L + \vec{F}_D + \vec{F}_S = 0, \quad [14]$$

where $\vec{F}_{Br}$ is the Brownian force acting on the, $\vec{F}_L$ is the Lorentz force, $\vec{F}_D$ is the drag force and $\vec{F}_S$ is the force due to the harmonic spring. Using a well known result from equilibrium statistical mechanics that the Brownian force $\vec{F}_i^{Br}$ can be written as $k_B T \nabla_i \ln P$, one obtains the following expression for velocity $\vec{\vartheta}_i$.

$$\vec{\vartheta}_i = \mathbf{\Gamma}_i^{-1} \big(- \nabla_i \phi + k_B T \nabla_i \ln P \big), \quad [15]$$

where the spring force on particle i is given as $-\nabla_i \phi$. Substituting above expression for velocity in Eq. (13), we get the following FPE equation:

$$\frac{\partial P}{\partial t} = k_B T \Big[\nabla_1 \cdot \mathbf{\Gamma_1}^{-1} \Big(\nabla_1 P + \frac{1}{k_B T} P \nabla_1 \phi \Big) + \nabla_2 \cdot \mathbf{\Gamma_2}^{-1} \Big(\nabla_2 P + \frac{1}{k_B T} P \nabla_2 \phi \Big) \Big]. \quad [16]$$

For zero rest length, $\nabla \phi$ can be written as

$$\nabla \phi = \mu \begin{bmatrix} 1 & 0 & -1 & 0 \\ 0 & 1 & 0 & -1 \\ -1 & 0 & 1 & 0 \\ 0 & -1 & 0 & 1 \end{bmatrix} \begin{bmatrix} x_1 \\ y_1 \\ x_2 \\ y_2 \end{bmatrix} = \mu \mathbf{K} \vec{X}, \quad [17]$$

where the operator ∇ stands for

$$\nabla = [\partial_{x_1}, \partial_{y_1}, \partial_{x_2}, \partial_{y_2}]^T. \quad [18]$$

The FPE can be arranged as

$$\frac{\partial P}{\partial t} = \nabla \cdot \Big[\boldsymbol{\mathcal{D}} \nabla P + \boldsymbol{\mathcal{G}} \vec{X} P \Big], \quad [19]$$

where

$$\boldsymbol{\mathcal{D}} = k_B T \begin{bmatrix} \mathbf{\Gamma}_1^{-1} & \mathbf{0} \\ \mathbf{0} & \mathbf{\Gamma}_2^{-1} \end{bmatrix}, \qquad \boldsymbol{\mathcal{G}} = \mu \begin{bmatrix} \mathbf{\Gamma}_1^{-1} & \mathbf{0} \\ \mathbf{0} & \mathbf{\Gamma}_2^{-1} \end{bmatrix} \mathbf{K}. \quad [20]$$

$\mathbf{0}$ is null block matrix of dimension 2×2. Equation 19 is a linear multivariate FPE that admits the following Gaussian solution:

$$P(\vec{X},t)=\frac{1}{4\pi^2\sqrt{\det(\mathbf{\Sigma})}}\exp\Big(-\frac{1}{2}\vec{X}^T\mathbf{\Sigma}^{-1}\vec{X}\Big), \tag{21}$$

where $\mathbf{\Sigma}$ satisfies the Lyapunov equation

$$\frac{d\mathbf{\Sigma}}{dt}=\mathbf{A}\mathbf{\Sigma}+\mathbf{\Sigma}\mathbf{A}^T+\mathbf{B}. \tag{22}$$

Here $\mathbf{A}$ is $-\mu\mathbf{G}$ and $\mathbf{B}$ is the symmetric part of the matrix $2\mathbf{D}$. $\mathbf{\Sigma}$ can be calculated as

$$\mathbf{\Sigma}=\int_0^t e^{-\mathbf{A}t}\boldsymbol{B}e^{-\mathbf{A}^Tt}=\begin{bmatrix}a & 0 & b & c\\ 0 & a & -c & b\\ b & -c & a & 0\\ c & b & 0 & a\end{bmatrix}. \tag{23}$$

Here

$$a=2D\tau_s\left(\frac{\kappa^2+1}{4}\left(1-e^{-4\frac{t}{\tau_m}}\right)+\frac{t}{\tau_s}-\kappa^2\left(1-e^{-2\frac{t}{\tau_m}}\right)\right), \tag{24}$$

$$b=2D\tau_s\left(\frac{\kappa^2-1}{4}\left(1-e^{-4\frac{t}{\tau_m}}\right)+\frac{t}{\tau_s}-\kappa^2\left(1-e^{-2\frac{t}{\tau_m}}\right)\right), \tag{25}$$

$$c=D\kappa\tau_s\left(-\frac{1}{2}\left(1-e^{-4\frac{t}{\tau_m}}\right)+\left(1-e^{-2\frac{t}{\tau_m}}\right)\right). \tag{26}$$

The MSD for the center of mass and the bond vector fluctuations can be computed easily as follows

$$\langle R_x^2\rangle=\langle R_y^2\rangle=\frac{1}{2}(a+b), \tag{27a}$$

$$\langle r_x^2\rangle=\langle r_y^2\rangle=2(a-b). \tag{27b}$$

3. Meandering Dimers : Derivation of Fokker Planck Equation

In this section, we derive the FPE for an arbitrary shaped particle assuming its translational drag tensor is $\mathbf{\Gamma}_t$ and rotational drag tensor is $\mathbf{\Gamma}_r$. This derivation is based on a general method discussed in Ref. (5). The particle carries two charges, q_1 and q_2, at a distance of $2r_0$. The two charges are equidistant from the center of mass. For a particle moving with an instantaneous velocity $\vec{\vartheta}$ and an angular velocity $\vec{\Omega}$, the Lorentz force $\vec{F}_L$ and the Lorentz torque $\vec{\mathcal{T}}_L$ are given as

$$\vec{F}_L=-2q^+B\mathbf{N}\vec{\vartheta}+2q^-Br_0\mathbf{NU}\vec{\Omega}, \tag{28a}$$

$$\vec{\mathcal{T}}_L=-2q^-Br_0\mathbf{UN}\vec{\vartheta}+2q^+Br_0^2\mathbf{UNU}\vec{\Omega}. \tag{28b}$$

The net force and the net torque acting on a dimer is zero in the overdamped regime. This straightforwardly yields

$$\left[\mathbf{\Gamma}_t+2q^+B\mathbf{N}\right]\vec{\vartheta}-2q^-Br_0\mathbf{NU}\vec{\Omega}-\vec{F}_{Br}=0, \tag{29a}$$

$$\left[\mathbf{\Gamma}_r-2q^+Br_0^2\mathbf{UNU}\right]\vec{\Omega}+2q^-Br_0\mathbf{UN}\vec{\vartheta}-\vec{\mathcal{T}}_{Br}=0. \tag{29b}$$

For a short hand notation, we define

$$\mathbf{M}_H=\mathbf{\Gamma}_t+2q^+B\mathbf{N}\quad\text{and}\quad\mathbf{M}_R=\mathbf{\Gamma}_r-2q^+Br_0^2\mathbf{UNU}.$$

To keep the calculations concise, we denote the term $2q^-Br_0$ as $\mathcal{K}$. We also use following results to write the Brownian forces and torques in terms of the probability density functions

$$\vec{F}_{Br}=k_BT\,\nabla\ln P. \tag{30a}$$

$$\vec{\mathcal{T}}_{Br}=k_BT\,\hat{\mathcal{R}}\ln P, \tag{30b}$$

where the rotation operator reads as $\hat{\mathcal{R}}=\partial/\partial\theta$ in polar coordinates.

The FPE describing the diffusion behaviour of the particle is of the form

$$\frac{\partial P}{\partial t}=\nabla\cdot\left[\vec{\vartheta}P\right]+\hat{\mathcal{R}}\cdot\left[\vec{\Omega}P\right], \tag{31}$$

where $\vec{\vartheta}P$ and $\vec{\Omega}P$ are the probability fluxes in the translational and orientational spaces, respectively. Substituting for $\vec{\vartheta}$ and $\vec{\Omega}$ from Eq. (29) in the above FPE, one obtains

$$\frac{\partial P}{\partial t}=k_BT\,\nabla\cdot\boldsymbol{\mathcal{M}}_H\left[\nabla P+\mathcal{K}\mathbf{NUM}_R^{-1}\hat{\mathcal{R}}P\right]+k_BT\,\hat{\mathcal{R}}\cdot\boldsymbol{\mathcal{M}}_R\left[\hat{\mathcal{R}}P-\mathcal{K}\mathbf{UNM}_{\mathrm{H}}^{-1}\nabla P\right]. \tag{31}$$

Here

$$\boldsymbol{\mathcal{M}}_H = \left[\mathbf{M}_\mathrm{H} + \mathcal{K}^2\mathbf{NUM}_\mathrm{R}^{-1}\mathbf{UN}\right]^{-1} \qquad \text{and} \qquad \boldsymbol{\mathcal{M}}_R = \left[\mathbf{M}_\mathrm{R} + \mathcal{K}^2\mathbf{UNM}_\mathrm{H}^{-1}\mathbf{NU}\right]^{-1}.$$

On the right hand side of the equation (31), the first term in the square brackets contains a contribution from rotational fluxes ($\propto \hat{\mathcal{R}}P$) whereas the second term contains a contribution from the translational fluxes ($\propto \nabla P$). In the absence of Lorentz force induced coupling between the translational and rotational degrees of freedom, these terms do not appear in the FPE as is usually the case. In the presence of Lorentz forces, the divergence of these extra terms is not necessarily zero, and hence they are important to quantify the evolution of probabilities.

The first term on the right hand side of the equation (31) indicates that if the orientational probability is not uniform, it may lead to extra probability fluxes that will influence the spatial diffusion. In this work, we circumvent this problem by focusing on the translational diffusion behavior of the particle when its rotational degree is relaxed to equilibrium, i.e., $\hat{\mathcal{R}}P = \vec{0}$. If particle is restricted on a flat 2D surface, orientation can be written as $\hat{u} = [cos\theta, sin\theta]^T$. Integrating the FPE over orientational degree of freedom θ, rotational divergence term in Eq. (31) goes to zero yielding the FPE

$$\frac{\partial P}{\partial t} = k_B T\, \nabla \cdot \left[\langle \boldsymbol{\mathcal{M}}_H\rangle_\theta \nabla P\right], \tag{32}$$

where $\boldsymbol{\mathcal{M}}_H$ is the orientation dependent diffusion tensor. Its average over all possible orientation is denoted as $\langle \boldsymbol{\mathcal{M}}_H\rangle_\theta$. Similarly, if the probability of a particle being at $\vec{R}$ is uniform and its orientation is fixed to $\hat{u} = 0$, then Eq. (31) reduces to

$$\frac{\partial P}{\partial t} = k_B T \hat{\mathcal{R}} \cdot \left[\boldsymbol{\mathcal{M}}_R \hat{\mathcal{R}} P\right]. \tag{33}$$

A. Rigid Dimers carrying arbitary charges. Consider a dimer carrying arbitrary charges $q_1 = q$ and $q_2 = xq$ on its ends. We decompose the charge distribution into sets of equal and opposite charges. On one end, the dimer carries $q^+ + q^-$ units charge and on the other $q^+ - q^-$ units. Here $q^+ = (1+x)q/2$ and $q^- = (1-x)q/2$. We define α and β as

$$\alpha = (1-x)\frac{\kappa}{2}, \qquad \beta = (1+x)\frac{\kappa}{2}. \tag{34}$$

Under the assumption that the orientational degree of freedom is relaxed to equilibrium, the diffusion tensor reads

$$\begin{aligned}\boldsymbol{\mathcal{D}}(\hat{u}) =& D_t \begin{bmatrix} \frac{1+\alpha^2}{1+\alpha^2+\beta^2} & \frac{\beta}{1+\alpha^2+\beta^2} \\ \frac{-\beta}{1+\alpha^2+\beta^2} & \frac{1+\alpha^2}{1+\alpha^2+\beta^2}\end{bmatrix} \\ &- D_t \frac{\alpha^2}{1+\alpha^2+\beta^2}\hat{\mathbf{u}}\hat{\mathbf{u}}.\end{aligned} \tag{34}$$

The above tensor implies that the diffusion of a dimer along its orientation is

$$\mathcal{D}_{\hat{u}} = \frac{1}{1+\alpha^2+\beta^2} D_t. \tag{35}$$

To understand the above result we refer to the dynamical description discussed in the main text. Consider an external force $\vec{F}$ that drags the dimer along its orientation $\hat{u}$. As discussed in the main text, a set of opposite charges of magnitude $|q^-|$ gives rise to a magnetic drag force. At the same time, a set of equal charges held on the dimer ($|q^+|$) induces a Lorentz force that curves the trajectory of the centre of mass of the dimer. As a consequence, the diffusion along the orientation reduces by a factor of $1/(1+\alpha^2+\beta^2)$.

Interestingly, the diffusion constant along the direction perpendicular to orientation is

$$\mathcal{D}_\perp = \frac{1+\alpha^2}{1+\alpha^2+\beta^2} D_t. \tag{36}$$

It follows that a set of q^- charges on the dimer ends decreases the curving effect if the rod is translated perpendicular to its orientation. If the force is only applied in the direction perpendicular to $\hat{u}$, charges with q^+ magnitude induce a Lorentz force that acts along the dimer's orientation. Because of the charges q^-, such a force experiences extra drag according to Eq. (25) in the main text. The curving effect, therefore, gets reduced. On considering the limit of both α and β going to ∞, it is immediately clear that diffusion orthogonal to the orientation does not vanish. This demonstrates that even the isotropic diffusion coefficient should not tend towards zero for arbitrarily high x. From Eq. (34), it automatically follows that the following must be the isotropic diffusion tensor.

$$\langle \boldsymbol{\mathcal{D}}_t\rangle = D_t \begin{bmatrix} \frac{1+\frac{\alpha^2}{2}}{1+\alpha^2+\beta^2} & \frac{\beta}{1+\alpha^2+\beta^2} \\ \frac{-\beta}{1+\alpha^2+\beta^2} & \frac{1+\frac{\alpha^2}{2}}{1+\alpha^2+\beta^2}\end{bmatrix} \tag{37}$$

Similarly, for a spatially homogeneous system, rotational diffusion coefficient is

$$\mathcal{D}_r = \frac{D_r}{1+\frac{\alpha^2}{1+\beta^2}}. \tag{38}$$

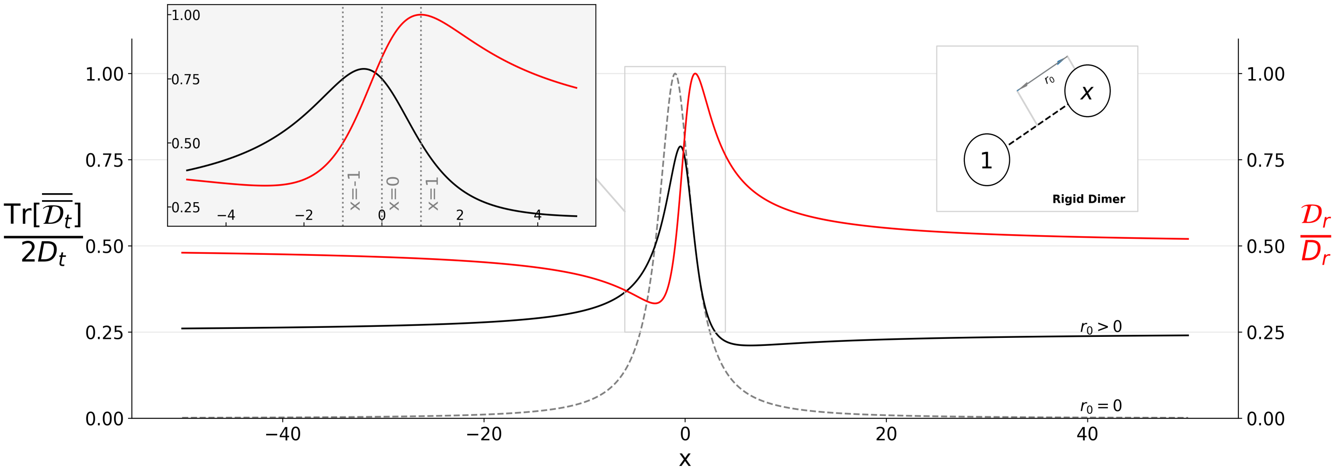

Fig. S1. In right inset, a rigid dimer of length $2r_0$ caries charge q on one end and charge xq on the other end. We plot isotropic translational diffusion (solid black line) and rotational diffusion (red line) of a dimer as a function of x. Dotted grey line shows the diffusion constant of a single particle carrying $q(1+x)$ charge. Unlike a single charged particle, even if x is arbitrarily high, the diffusion coefficient of the dimer does not vanish. Rather it tends to $D_t/4$. (Grey inset) Dimer's translational diffusion is maximum at $x = \frac{3}{\kappa^2} - \sqrt{\frac{9}{\kappa^4} + \frac{2}{k^2} + 1}$ instead of $x = -1$ and achieves minimum for $x = \frac{3}{\kappa^2} + \sqrt{\frac{9}{\kappa^4} + \frac{2}{k^2} + 1}$ The rotational diffusion coefficient is unaffected if $x = -1$ and is minimum for $x = -\left(1 + \frac{2}{\kappa^2}\right)$

As we showed in previous sections, for an isotropic configuration, spatial diffusion dynamics of a centre of mass of a dimer carrying equal charges of magnitude q is equivalent to the diffusion of a particle carrying $2q$ charge. Similarly, a dimer carrying opposite charges has spatial diffusion qualitatively similar to an uncharged particle. Naively one may think when $x = -1$, dimer has maximum mobility and that it decreases as one increases x to arbitrarily high values. But as Fig S1 depicts, this is not the case. These non-trivial results clearly show that charge separation alters the behaviour of a dimer permanently and even qualitatively one can not treat a dimer as a single particle carrying some equivalent charge.

4. Diffusion of charged dimers in 3D

Here we calculate diffusion tensors that characterize the motion of a rigid dimer in three dimensions. The direction of the magnetic field is set to $\hat{n} = [0, 0, 1]^T$. We focus on the special cases of equal and opposite charges dimer. The diffusion tensor for a dimer carrying equal charges *i.e.* $\alpha = 0$ reads

$$\mathbf{D}_r^+ = \begin{bmatrix} \frac{1}{1+\beta^2} & \frac{-\beta}{1+\beta^2} & 0 \\ \frac{\beta}{1+\beta^2} & \frac{1}{1+\beta^2} & 0 \\ 0 & 0 & 1 \end{bmatrix} \quad [39]$$

and its rotational diffusion tensor reads

$$\mathbf{D}_r^+ = \frac{k_B T}{\gamma_r}\left(\mathbf{I} + \frac{(\hat{n}\cdot\hat{u})^2\beta^2}{1+(\hat{n}\cdot\hat{u})^2\beta^2}\mathbf{U}^2 - \frac{(\hat{n}\cdot\hat{u})\beta}{1+(\hat{n}\cdot\hat{u})^2\beta^2}\mathbf{U}\right). \quad [40]$$

In case of a dimer with opposite charges, i.e., $\beta = 0$, its mobility tensor reads

$$\boldsymbol{\mathcal{M}}_H = \begin{bmatrix} \frac{1+\alpha^2(1-u_x^2)}{u_z^2\alpha^4+u_z^2\alpha^2+\alpha^2+1} & \frac{-u_x u_y \alpha^2}{u_z^2\alpha^4+u_z^2\alpha^2+\alpha^2+1} & 0 \\ \frac{-u_x u_y \alpha^2}{u_z^2\alpha^4+u_z^2\alpha^2} & \frac{1+\alpha^2(1-u_y^2)}{u_z^2\alpha^4+u_z^2\alpha^2+\alpha^2+1} & 0 \\ 0 & 0 & 1 \end{bmatrix}. \quad [41]$$

Note, that for $u_z = 0$, the above tensor reduces to the tensor mentioned in the main text (Eq. (22)). Surprisingly, even for $u_z = 1$, the mobility in the x-y plane still gets reduced by $1/(1+\alpha^2)$. One can calculate the rotational diffusion tensor assuming that the system is homogeneous, i.e., $P(\vec{R}, \hat{u}, t) = P(\hat{u}, t)$ as

$$\mathbf{D}_r^- = \frac{k_B T}{\gamma_r}\begin{bmatrix} \frac{1+\frac{u_x^2 u_z^2\alpha^4}{1+\alpha^2}}{1+u_z^2\alpha^2} & \frac{\frac{u_x u_y u_z^2\alpha^4}{1+\alpha^2}}{1+u_z^2\alpha^2} & \frac{u_x u_z\alpha^2}{1+\alpha^2} \\ \frac{\frac{u_x u_y u_z^2\alpha^4}{1+\alpha^2}}{1+u_z^2\alpha^2} & \frac{1+\frac{u_y^2 u_z^2\alpha^4}{1+\alpha^2}}{1+u_z^2\alpha^2} & \frac{u_y u_z\alpha^2}{1+\alpha^2} \\ \frac{u_x u_z\alpha^2}{1+\alpha^2} & \frac{u_y u_z\alpha^2}{1+\alpha^2} & \frac{1+u_z^2\alpha^2}{1+\alpha^2} \end{bmatrix}. \quad [42]$$

5. Diffusion of rod like Particles

We consider a rod like particle with a friction coefficient $\gamma_{||}$ along its orientation and $\gamma_{\perp}$ along the direction perpendicular to the orientation. We assume the relation $\gamma_{||} = \varepsilon\gamma_{\perp}$. The friction tensor reads

$$\mathbf{\Gamma}_t = \mathbf{I} + (\varepsilon - 1)\hat{\mathbf{u}}\hat{\mathbf{u}}. \quad [43]$$

The length of the particle is $2r_0$ and has a rotational drag coefficient γ_r. Classic bead rod model can be considered as one of the special cases where $\varepsilon = 1/2$. $\varepsilon = 1$ is a limiting case of a spherical particle. We use the following relation Eq. (43) to calculate the inverses in Eq. (31):

$$[\mathbf{I} - m\hat{\mathbf{u}}\hat{\mathbf{u}} + n\mathbf{N}]^{-1} = \frac{1}{(m-1) - n^2[1 - m(\hat{n}\cdot\hat{u})^2]}\Big[(m - 1 - n^2)\mathbf{I} - m\hat{\mathbf{u}}\hat{\mathbf{u}} - n^2\mathbf{N}^2 + n\mathbf{N} - mn(\hat{n}\cdot\hat{u})\mathbf{U}\Big]. \quad [43]$$

We focus on the particular case of a single rod performing diffusion in a 2D plane. For this case, the orientation dependent diffusion tensor of the rod reads

$$\boldsymbol{\mathcal{D}}(\hat{u}) = \frac{k_BT}{\gamma_\perp}\begin{bmatrix} \frac{\varepsilon+\alpha^2}{\varepsilon+\alpha^2+\beta^2} & \frac{\beta}{\varepsilon+\alpha^2+\beta^2} \\ \frac{-\beta}{\varepsilon+\alpha^2+\beta^2} & \frac{\varepsilon+\alpha^2}{\varepsilon+\alpha^2+\beta^2}\end{bmatrix} - \frac{k_BT}{\gamma_\perp}\frac{\varepsilon - 1 + \alpha^2}{\varepsilon + \alpha^2 + \beta^2}\hat{\mathbf{u}}\hat{\mathbf{u}}. \quad [44]$$

The rotational diffusion constant is given as

$$\mathcal{D}_r = \frac{1}{1 + \frac{\alpha^2}{(\varepsilon+\beta^2)}}. \quad [45]$$

6. Simulation Results

A. General case : Flexible Rouse dimer with a non-zero rest length. So far, we focused on the special cases of flexible dimer of zero rest length and rigid dimer of finite rest length. We now study the more general case of a charged Rouse dimer with a non-zero rest length. The following coupled overdamped Langevin equations describe the dynamics of a charged dimer:

$$\mathbf{\Gamma}_1\frac{d\vec{r}_1}{dt} = -k\left(1 - \frac{l_0}{|\vec{r}_1 - \vec{r}_2|}\right)(\vec{r}_1 - \vec{r}_2) + \vec{\eta}_1(t), \quad [46]$$

$$\mathbf{\Gamma}_2\frac{d\vec{r}_2}{dt} = -k\left(1 - \frac{l_0}{|\vec{r}_1 - \vec{r}_2|}\right)(\vec{r}_2 - \vec{r}_1) + \vec{\eta}_2(t). \quad [47]$$

We focus on a Rouse dimer carrying opposite charges, i.e., $q_1 = -q_2 = q$ such that $\mathbf{\Gamma}_1 = \gamma(\mathbf{I} + \kappa\boldsymbol{N})$ and $\mathbf{\Gamma}_2 = \gamma(\mathbf{I} - \kappa\boldsymbol{N})$. Also, $\alpha = \kappa$ and $\beta = 0$ (see Eq. (34)). We choose the rest length to be one order of magnitude higher than the length scale of typical spring fluctuations, i.e., $l_0 = 10\sqrt{k_BT/k}$. Since the magnetic field is spatially constant and we are only interested in the position statistics of the dimer, we can safely ignore the non-Gaussian nature of the noise $\vec{\eta}_i$. We use standard Euler-Maruyama numerical integration scheme to integrate the above stochastic differential equation. Brownian dynamics simulations reveal that similar to the case of the flexible Rouse dimer (discussed in the main text), the center of mass of the dimer exhibits normal diffusion both at short and long times but with different diffusion coefficients. At short times, the center of mass of the dimer diffuses with the diffusion constant $D/(1+\kappa^2)$. In the crossover, the center of mass accelerates until its diffusion constant becomes equal to $(1+0.5\alpha^2)D/(1+\alpha^2)$. This diffusion constant is of a rigid Rouse dimer which carries opposite charges on its ends. This validates our theoretical prediction that diffusion of charged dimer under magnetic field is anisotropic.

B. Polymer-like systems. In this section, we present simulation results for long charged chains subjected to an external magnetic field. A freely diffusing Rouse chain, a frequently used model in polymer physics, is composed of multiple Brownian beads connected by harmonic springs. Consider a rouse chain made up of N beads, each carrying a charge of either $+q$ or $-q$ units. We do not take into consideration the excluded volume interactions between these beads. An overdamped Langevin equation that describes the dynamics of the charged Rouse chain is given as

$$\mathbf{\Gamma}_i\frac{d\vec{r}_i}{dt} = -k(2\vec{r}_i - \vec{r}_{i+1} - \vec{r}_{i-1}) + \vec{\eta}_i(t), \quad [48]$$

where $\mathbf{\Gamma}_i = \gamma(\mathbf{I} + \kappa_i\boldsymbol{N})$. Here $i = 2, 3, .., N-1$. If the chain is open ended, the Langevin equations for the bead labeled $i = 0$ and $i = N$ are

$$\mathbf{\Gamma}_1\frac{d\vec{r}_1}{dt} = -k(\vec{r}_1 - \vec{r}_2) + \vec{\eta}_1(t) \quad \text{and} \quad \mathbf{\Gamma}_N\frac{d\vec{r}_N}{dt} = -k(\vec{r}_N - \vec{r}_1) + \vec{\eta}_N(t), \quad [49]$$

respectively. Whereas, if the beads are connected in a ring topology, the above Langevin equations read

$$\mathbf{\Gamma}_1\frac{d\vec{r}_1}{dt} = -k(2\vec{r}_1 - \vec{r}_2 - \vec{r}_{2N}) + \vec{\eta}_1(t) \quad \text{and} \quad \mathbf{\Gamma}_N\frac{d\vec{r}_N}{dt} = -k(2\vec{r}_N - \vec{r}_1 - \vec{r}_{N-1}) + \vec{\eta}_N(t). \quad [50]$$

We study the dynamics of Rouse chains (6, 7) and rings which are overall charge neutral. We focus on two specific charge distributions: alternating charges and block charges. As the name suggests, in the first case the chain (ring) is composed of

(a) Alternating positively and negatively charged beads in ring topology

(b) Block distributed positively and negatively charged beads in ring topology

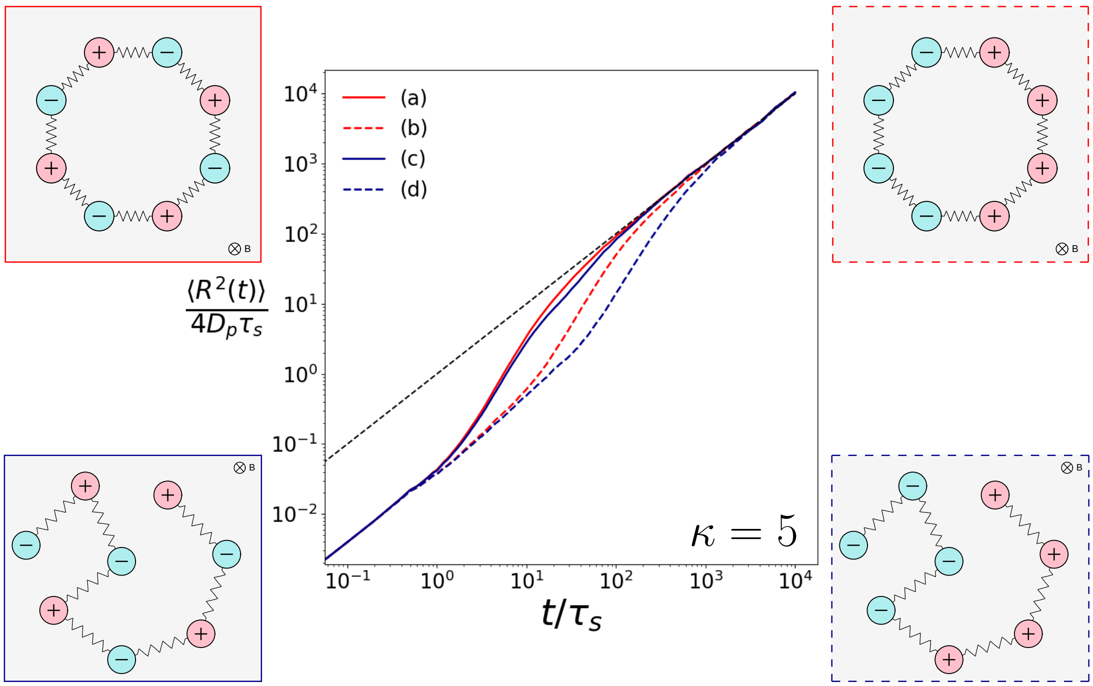

(c) Alternating positively and negatively charged beads in open chain

(d) Block distributed positively and negatively charged beads in open chain

Fig. S2. Topology and charge distribution: The plot displays normalised mean squared displacement (MSD) of the center of mass of a flexible Rouse chain (ring) consisting of $N = 8$ beads for $\kappa = 5$. Two different charge distributions and two different topologies are considered as shown above in the schematics. For all cases, the center of mass initially diffuses with diffusion constant $D_p/(1+\kappa^2)$, followed by a crossover where it undergoes acceleration. Here, $D_p = D_0/N$ is the diffusion constant of a chain with N beads. The length and the onset of the crossover time window depends on charge distribution as well as topology. For block distributed charges, the crossover is significantly wider and shifted forward in time compared to the crossover for a polymer carrying alternating charges. The rings show accelerated dynamics for a shorter time interval compared to a chain carrying an equivalent charge distribution.

alternating positively and negatively charged beads. In the second case, the chain (ring) has blocks of positively and negatively charged beads such that one half of the chain (ring) is positively charged and other is negatively charged.

Brownian dynamics simulations show that charged chains, irrespective of their charge distribution, initially diffuse with a diffusion constant $D_p/(1+\kappa^2)$. Here, $D_p = D_0/N$ is the diffusion constant of a chain with N beads. Rings and chains with same charge distributions show similar behavior. For both topologies, when the charge is distributed in alternating fashion, the dynamics are very similar to that of a Rouse dimer (Fig. S2(a) and (c)). However, in the case of block distributed charges, the onset of enhancement as well as the crossover time increases dramatically (Fig. S2(b) and (d)). The width of crossover for chains with random charge distribution is in between the two extreme cases of alternating charges and block charges (Fig. S3). For a given length, the chain topology with block distributed charges exhibits the longest crossover time. In the main text, we show that the crossover time scales as N^2, which corresponds to the slowest mode of relaxation (Rouse time) of the polymer chain. For each chain, we measure the start and the end of its crossover period by measuring the times at which the chain's normalised MSD crosses the lines $y = 0.9x$ and $y = 1.1x/(1+\kappa^2)$, respectively.

References

1. HD Vuijk, JM Brader, A Sharma, Anomalous fluxes in overdamped Brownian dynamics with Lorentz force. *J. Stat. Mech. Theory Exp.* **2019**, 063203 (2019).
2. HM Chun, X Durang, JD Noh, Emergence of nonwhite noise in Langevin dynamics with magnetic Lorentz force. *Phys. Rev. E* **97**, 032117 (2018).
3. R Pawula, Approximation of the linear Boltzmann equation by the Fokker-Planck equation. *Phys. review* **162**, 186 (1967).
4. I Abdoli, HD Vuijk, JU Sommer, JM Brader, A Sharma, Nondiffusive fluxes in a Brownian system with Lorentz force. *Phys. review E* **101**, 012120 (2020).
5. JK Dhont, WJ Briels, Rod-like Brownian particles in shear flow. *Soft Matter: Complex Colloidal Suspens.* (2004).
6. PE Rouse Jr, A theory of the linear viscoelastic properties of dilute solutions of coiling polymers. *The J. Chem. Phys.* **21**, 1272–1280 (1953).

7. M Doi, SF Edwards, SF Edwards, *The theory of polymer dynamics.* (oxford university press) Vol. 73, (1988).

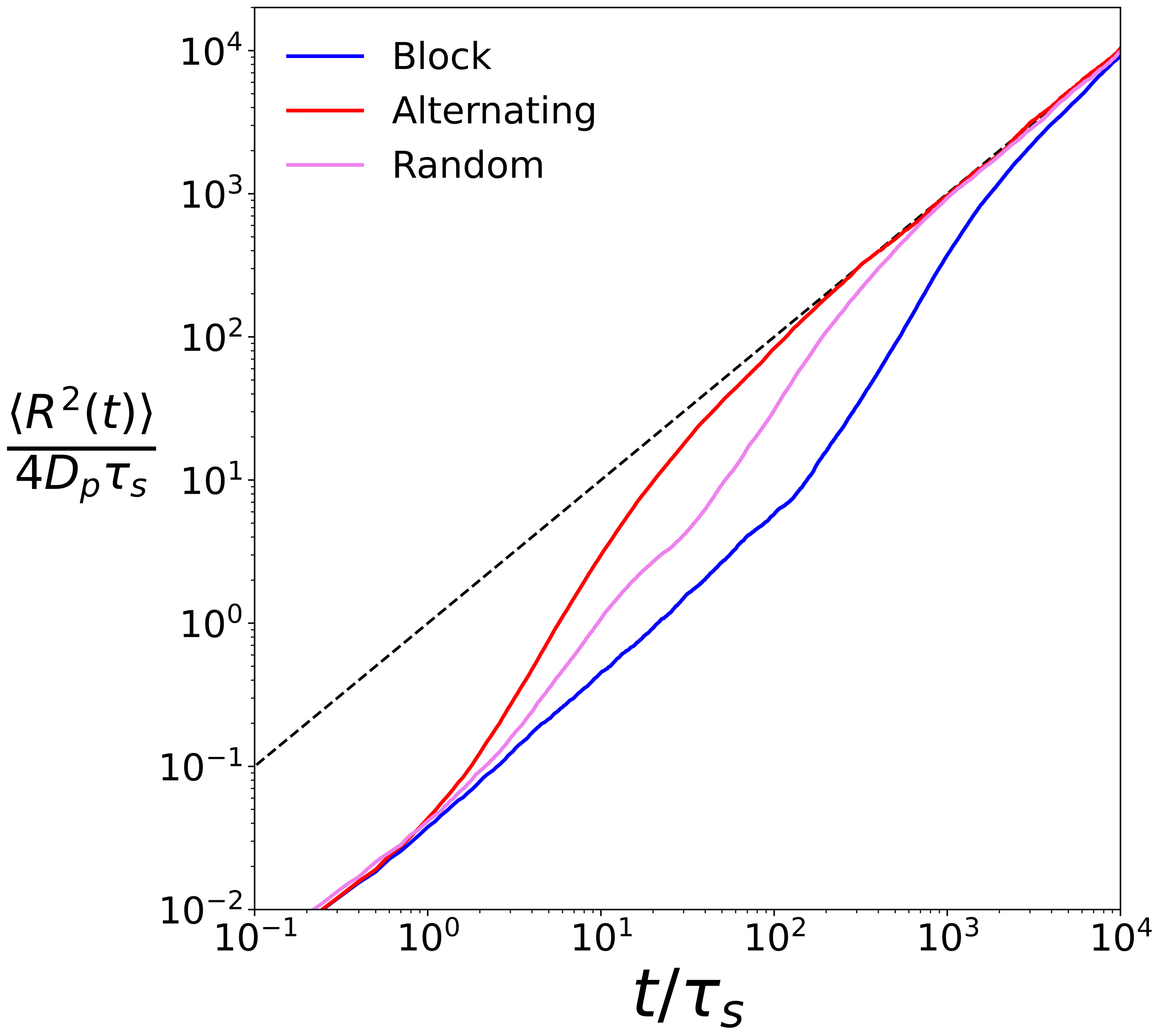

Fig. S3. Normalised mean squared displacement of the center of mass of a flexible charged Rouse chain consisting of $N = 16$ beads for $\kappa = 5$. The chains are overall charge neutral but have different charge distributions. For all cases, the center of mass initially diffuses with diffusion constant $D_p/(1+\kappa^2)$, followed by a crossover where it undergoes acceleration. Here, $D_p = D_0/N$ is the diffusion constant of a chain with N beads. A chain that carries random charges shows diffusive behavior between the extreme cases of block distributed charges and alternating charges.
